# Evaluation of Resilience and Mental Health in the “Post-Pandemic Era” among University Students: Protocol for a Mixed-Methods Study

**DOI:** 10.3390/ijerph21070825

**Published:** 2024-06-25

**Authors:** Natasja Kudzai Magorokosho, Alexandros Heraclides, Eleonora Papaleontiou-Louca, Maria Prodromou

**Affiliations:** 1 School of Sciences, European University Cyprus, Nicosia 2404, Cyprus; 2 School of Humanities, Social & Education Sciences (Psychology), European University Cyprus, Nicosia 2404, Cyprus; e.papaleontiou@euc.ac.cy

**Keywords:** COVID-19 pandemic era, university students, mental health, COVID-19, post-pandemic, public health

## Abstract

Background: The mental well-being of university students has been a growing concern in Public Health and has been exacerbated by the COVID-19 pandemic. The pandemic (including the post-pandemic era) introduced and exacerbated a variety of potential stressors for vulnerable individuals and communities, resulting in an increase in mental health issues among university students. Resilience, as a process, is the ability of a system to adapt and grow in the face of adversity. This is a crucial aspect to consider when examining the coping of university students in critical situations such as COVID-19. Objective: This study aims to assess the association between resilience and mental health outcomes among university students in Cyprus during the post-COVID-19 pandemic era. Methods: A parallel embedded mixed methods research design will be utilized to assess resilience, measured by the Conner–Davidson Resilience Scale (CD-RISC), the COVID-19 Impact Scale (CIS) and mental health outcomes measured by the Symptom Checklist 90-Revised (SCL 90-R), during the COVID-19 post-pandemic era (January 2022–July 2024) among university students in the Republic of Cyprus. The study will be conducted in two stages: a pilot study followed by the main study. Quantitative data will be collected through a web-based survey, while qualitative data will be obtained through in-person focus groups designed to capture participants’ experiences. Participants will be recruited using a proportional quota sampling approach to achieve representativeness based on predefined demographics. The study protocol has been approved by the Cyprus Bioethics Committee (approval no: EEC/EP/2023/31). Discussion: This study is expected to broaden our understanding of the intricate interactions between the COVID-19 pandemic’s impact, resilience, and mental health outcomes. The focus on university students’ psychological wellbeing is consistent with the call by the WHO to focus on mental health (World Health Organization, 2019).

## 1. Introduction

### 1.1. Background

The mental health of the student population has been a major concern for several years [1]. Researchers have found that major pandemics (e.g., the Spanish flu) exacerbate mental health outcomes of university students. In the period 2020–2022, the peak of the COVID-19 pandemic, most university students were forced to switch from in-person to virtual (online) class arrangements [2,3,4,5]. In addition, strict lockdowns and mandated isolations were put in place to mitigate the spread of COVID-19; however, this significantly compromised social networks across the global population [6,7,8]. These mitigating measures restricted social gatherings, particularly among younger people, and contributed to a lack of social and community assistance—which are both critical to mitigating and recovering from possible mental health issues [3,9,10]. Notably, during the COVID-19 pandemic, increases in the onset and intensification of stress, sleeplessness, fear, anger, anxiety, and depressive symptoms were observed among university students. These changes have been attributed to the sudden shift in public health regulations, such as social distance and self-isolation mandates [4,11,12].

Numerous studies from different countries have reported on students’ thoughts and reactions to unpredictable and life-threatening situations such as COVID-19 pandemic [10,13,14,15]. According to a Chinese survey, the COVID-19 pandemic made 25% of university students fearful [16]. In an online poll of Turkish university students, 38% said they feared contracting the COVID-19 virus [2]. In addition, studies conducted in the European Union reported that the COVID-19 pandemic has caused a 60% increase in stress and anxiety among university students in France, Spain, and Poland [17,18,19]. Around 40–60% of students at universities throughout Germany’s federal states reported higher levels of mental stress, loneliness, and worry about the future [19]. Conversely, however, about 17% of Bavarian students said the COVID-19 pandemic had reduced their mental stress [19]. Considering the complexity of these data, it is evident that the COVID-19 pandemic had a complex effect on university students’ mental health that differs among groups and geographical areas. The wide range of experiences and responses among students emphasizes the need for focused and flexible mental health interventions.

Social support has been shown to mediate the relationship between perceived stress and mental health for many years. Social support comes from relationships with others, groups, and the community [20,21]. Individuals often seek help from family and social networks after experiencing trauma. During the COVID-19 pandemic, the importance of social support and resilience has been heightened as individuals face unprecedented challenges and uncertainty [22,23]. Understanding the role of ecological assets, such as social support, in mediating the impact of crisis on mental health is crucial in providing effective interventions and support for university students.

Data show that social support not only serves as a buffer against the negative effects of stress and trauma but also plays a significant role in fostering resilience [23]. It encompasses emotional, instrumental, informational, and appraisal support from various sources, including family, friends, peers, and community networks.

In the context of the COVID-19 pandemic, the absence of social support could have been a factor in university students’ ability to cope with their academic and social lives. Exploring the different types of social support and how they interact with individual coping strategies can provide valuable insights into the resilience and mental well-being of students during this challenging time.

There is a dearth of insight on university students’ lived experiences during the COVID-19 pandemic. Moreover, there is a lack of evidence on the long-term impacts of the pandemic on students’ mental health, including difficulties in re-establishing social connections and networks, and the mental health impact of COVID-19. Therefore, this study aims to broaden our understanding of the intricate interactions between the COVID-19 pandemic’s impact, resilience, and the mental health outcomes of university students, which advances scientific and clinical practice knowledge. Although other studies, including those by [24,25,26,27] have looked at the relationship between resilience and mental health among university students, this study intends to provide several unique contributions in the context of COVID-19. That is its focus on the resilience and mental health of university students in the post-pandemic era, in contrast to earlier research that focused on pre- or early pandemic times [10,26,28]. In addition, the study will focus on the long-term consequences of the crisis on resilience and mental health outcomes by taking into consideration the unique difficulties and pressures that university students encountered in the wake of the COVID-19 pandemic. By investigating the impact of the pandemic on university students’ resilience and mental health, this research work seeks to close the gaps in the body of existing material [26,29]. The study explores specific ways that a global crisis, like the COVID-19 pandemic, might impact university students’ psychological health. This study examines the moderating and mediating factors that affect the relationship between the pandemic’s impact, mental health, and resilience. Furthermore, the study offers a nuanced analysis of how individual and environmental factors interact to affect outcomes through the rigorous investigation of variables including socioeconomic status, social support, and demographic attributes.

### 1.2. Conceptual Framework

The theory driving the current study is the Bronfenbrenner socio-ecological epistemologies. This theory recognizes that health outcomes and health behaviors are (1) subject to various levels of impact, (2) influenced by the individual’s environment, and (3) a result of interactions between the individual and their environment [30,31,32]. According to this perspective, individuals are embedded within a series of interconnected systems, ranging from the microsystem (individual interactions and experiences) to the macrosystem (broader cultural and societal influences). The framework emphasizes the dynamic and reciprocal nature of the interactions between the individual and their environment, highlighting the importance of understanding how contextual factors may contribute to health outcomes, in our case, mental health outcomes.

By using this approach to analyze university students both during and after the COVID-19 pandemic, we may better understand the interconnectedness of the COVID-19 pandemic and mental health outcomes of university students. The fundamental focus is the immediate settings in which students engage with one another, including their connections with teachers, peers, family, and friends [31]. The COVID-19 pandemic has impacted university students’ mental health and academic performance by shifting their immediate setting interactions to virtual platforms, thereby affecting the quality and nature of support and engagement and necessitating a deeper understanding of these shifts [33,34,35]. The microsystems in relation to the university students, focuses on the linkages between various microsystems, such as the link between family life and school experiences. For university students, the pandemic exacerbated the interplay between their academic obligations and family relationships [2,33,36,37]. In navigating these networked contexts, educational institutions’ support systems and peer networks become more important. The mesosystem is the broader context which includes external factors that have an indirect impact on the individual, such as university policies, government legislation, and media depictions [11]. The COVID-19 pandemic caused considerable modifications in university operations, including distant learning mandates and rearranged academic timetables. The exosystemic elements have a significant impact on students’ educational experiences and access to resources. This level includes broad societal and cultural values, conventions, and beliefs. The pandemic exposed, and in many cases worsened, existing socioeconomic inequities and cultural attitudes toward mental health and resilience [38,39,40]. Understanding how these broader socioeconomic influences affect university students’ coping methods and access to resources is critical for tackling systematic disparities. The chronosystem is the dimension that incorporates the element of time, emphasizing the impact of life transitions and historical events. The COVID-19 pandemic is a watershed moment in the timeline, affecting the life paths of university students. The long-term impact of these disturbances on resilience, mental health, and academic performance are critical areas of research [25]. Using Bronfenbrenner’s socio-ecological model, this study seeks to investigate the complex interplay between university students’ settings and their mental health and resilience in the post-pandemic period. Understanding these dynamics is critical for designing innovative therapies and support systems that meet university students’ unique needs during this unprecedented period. This approach helps in comprehending how different interconnected systems affect an individual’s growth and wellbeing, particularly in designing innovative interventions and support systems tailored to university students’ needs during this unprecedented period.

Variables of interest and how they connect with the conceptual framework are as follows:

i. COVID-19 Impact

This variable intersects with the exosystem level, reflecting broader environmental influences such as governmental responses, media discourse, and societal attitudes towards public health crises. Understanding the lingering effects of the pandemic on university students is paramount for developing targeted interventions and support systems. The impact of COVID-19 encompasses various aspects such as disruptions to education, financial strain, loss of social connections, increased stress, and mental health challenges [35,41,42]. Understanding the pandemic’s long-term repercussions, including disruptions in educational institutions, financial burden, and increased stress, is critical for developing tailored interventions and support networks for university students.

ii. Mental health outcomes:

Mental health outcomes are influenced by factors across multiple levels of the socio-ecological model, encompassing individual characteristics, interpersonal relationships, institutional support, and societal norms and values. Mental health outcomes among university students have been a growing concern even before the pandemic, but the crisis has exacerbated existing challenges and introduced new stressors [43,44,45]. The pandemic exacerbated pre-existing mental health issues, highlighting the importance of monitoring and addressing these consequences to improve academic performance and overall university student’s well-being.

iii. Resilience:

Resilience emerges from the interplay between individual characteristics and environmental contexts, highlighting the importance of coping mechanisms, social support networks, and access to resources in navigating adversity [46,47]. Resilience, the ability to bounce back from adversity, is a crucial protective factor in coping with the challenges posed by the pandemic and its aftermath [48]. Assessing resilience levels among university students can inform interventions aimed at fostering adaptive coping strategies, enhancing social support networks, and promoting mental health and academic success [49]. Building resilience can empower students to navigate uncertainty, setbacks, and transitions more effectively. Individual attributes and environmental factors combined to shape resilience. This variable appears in both the microsystem and mesosystem. The ability to recover from setbacks is critical in dealing with pandemic-related issues. Assessing resilience levels might help guide programs that promote adaptive coping methods and strengthen social support networks.

iv. Demographic Factors and Socio-Economic Status:

Demographic factors and socioeconomic status are variables that reflect larger societal and structural influences on students’ prospects and experiences. These variables are linked via the macrosystem and exosystem. Understanding how age, gender, ethnicity, and socioeconomic background influence post-pandemic outcomes is critical for encouraging equitable interventions and eliminating resource gaps [50,51]. Addressing disparities in access to resources, opportunities, and support systems is essential for promoting inclusive and equitable recovery efforts.

v. Social support:

Social support from family, friends, and the community is critical in mitigating the negative consequences of stress and hardship [22,23,52]. This variable appears in Bronfenbrenner’s socio-ecological model at the microsystem and mesosystem levels. Strengthening social support networks is crucial for increasing resilience and mental health among university students [27]. Improving access to counseling and mental health services can foster a supportive environment that promotes student well-being.

The COVID-19 pandemic has had a significant impact on university students’ mental health, resilience, and overall well-being. Examining these specific variables in the post-pandemic era is critical for several reasons, including the mental health crisis among university students. The pandemic has aggravated preexisting mental health issues among university students, resulting in higher rates of depression, anxiety, and stress. Understanding these consequences is critical to offering prompt interventions and assistance. Building resilience is critical for university students as they handle the ongoing uncertainty and challenges offered by the pandemic. Identifying variables that boost resilience might help guide initiatives for students’ academic and personal lives [53] 25 June 2024 10:55:00 A.M. The pandemic has highlighted and frequently exacerbated social inequities [54]. Examining the impact of demographics and socioeconomic status can aid in the development of equitable support networks for marginalized and disadvantaged students. Social support networks are crucial to student well-being. Improving these networks can assist to offset the negative effects of the pandemic and foster a supportive academic environment. This study aims to inform policymakers, educators, and healthcare professionals about the effects of the pandemic on university students’ mental health and resilience, allowing them to develop effective interventions and support systems tailored to the unique needs of students in the post-pandemic era.

### 1.3. Project Description

This study will assess the association between resilience and mental health outcomes among university students in Cyprus following the COVID-19 pandemic era, which is the period between 2022 and 2024. The overreaching scope of the proposed research study is to investigate mental health outcomes among university students in the post-pandemic era, including the psychological impact of COVID-19 and identify major determinants of these outcomes, such as socioeconomic factors and psychological resilience and related mediators, such as social support, while integrating the lived experiences of this population.

#### 1.3.1. Project Aims

Our primary aims are as follows:

Aim 1: To determine the association between the COVID-19 pandemic impact and mental health outcomes among university students during the post-pandemic era, in the Republic of Cyprus.

Aim 2: To investigate the association between COVID-19 impact and resilience of university students during the post-pandemic era.

Aim 3: To investigate the association between resilience and mental health outcomes among university students in Cyprus during the post-pandemic era.

Aim 4: To investigate resilience as a mediator/moderator between COVID-19 impact and mental health outcomes.

The above aims represent the overarching research questions that the study seeks to address, while the secondary aims that follow delve into specific aspects and factors related to the primary aims, providing additional insights, and understanding into the broader research questions:

Aim 5: To assess how demographic factors (e.g., age, gender, marital status) and socio-economic status (education, income, social class) moderate the association between COVID-19 impact and mental health outcomes.

Aim 6: To assess how social support moderates the association between COVID-19 impact and mental health outcomes.

Aim 7: To assess how demographic factors (e.g., age, gender, marital status) and socio-economic status (education, income, social class) moderate the association between COVID-19 impact and resilience.

Aim 8: To assess how social support moderates the association between COVID-19 impact and resilience.

Aim 9: To assess how demographic factors (e.g., age, gender, marital status) and socio-economic status (education, income, social class) moderate the association between resilience and mental health outcomes.

Aim 10: To assess how social support moderates the association between resilience and mental health outcomes.

Aim 11: To explore university students’ lived experiences during the COVID-19 pandemic, specifically resource change, as well as emotional and cognitive experiences.

#### 1.3.2. Study Hypothesis

Aim 1 Hypothesis: A significant association exists between the impact of the COVID-19 pandemic and adverse mental health outcomes during the post-pandemic era among university students in the Republic of Cyprus, with higher pandemic impact correlating with greater mental health challenges.

Aim 2 Hypothesis: A significant negative association exists between the impact of COVID-19 and resilience among university students in Cyprus during the post-pandemic era, suggesting that greater pandemic impact is linked with lower levels of resilience.

Aim 3 Hypothesis: Higher levels of resilience among university students in Cyprus during the post-pandemic era are associated with better mental health outcomes, indicating a positive correlation between resilience and mental well-being.

Aim 4 Hypothesis: Given an exposure to a major public health hazard, such as the COVID-19 pandemic, do individuals with higher resilience scores, cope better and are consequently protected from adverse health outcomes compared to those with low resilience scores.

Aim 5 Hypothesis: Demographic factors (e.g., age, gender, marital status) and socio-economic status (education, income, social class) moderate the association between COVID-19 impact and mental health outcomes among university students in Cyprus during the post-pandemic era, such that certain demographic groups and lower socio-economic strata will exhibit stronger associations between pandemic impact and mental health challenges.

Aim 6 Hypothesis: Social support moderates the association between COVID-19 impact and mental health outcomes among university students in Cyprus during the post-pandemic era, with higher levels of social support buffering against the negative effects of pandemic impact on mental well-being.

Aim 7 Hypothesis: Demographic factors and socio-economic status moderate the association between COVID-19 impact and resilience among university students in Cyprus during the post-pandemic era, indicating that certain demographic groups and lower socio-economic strata exhibit lower levels of resilience in response to pandemic-related stressors.

Aim 8 Hypothesis: Social support moderates the association between COVID-19 impact and resilience among university students in Cyprus during the post-pandemic era, with higher levels of social support buffering against the negative effects of pandemic impact on resilience.

Aim 9 Hypothesis: Demographic factors such as gender, age, etc., and socio-economic status moderate the association between resilience and mental health outcomes among university students in Cyprus during the post-pandemic era, suggesting that certain demographic groups and lower socio-economic strata and exhibit a lower degree of influence of resilience on mental well-being.

Aim 10 Hypothesis: Social support moderates the association between resilience and mental health outcomes among university students in Cyprus during the post-pandemic era, with higher levels of social support enhancing the protective effect of resilience on mental well-being.

Aim 11 Hypothesis: University students in Cyprus will report varied lived experiences during the COVID-19 pandemic, with specific demographic and socioeconomic groups reporting higher changes in resources, emotional responses, and cognitive processes, reflecting the multifaceted impact of the pandemic on their lives.

## 2. Methods

### 2.1. Study Design

The study will utilize a cross-sectional parallel embedded mixed-methods research design; data will be collected concurrently [55].

Quantitative data will be collected via a web-based questionnaire using Google Forms. Participating universities will distribute an email with the invitation to the study, to the enrolled university students. Interested individuals can then complete a 25 min survey and access the study by providing digital informed consent before proceeding. At the end of the survey, participants will be asked if they want to participate in a focus group, which would delve deeper into exploring individual emotional experiences during COVID-19.

Qualitative data will be collected via in-person focus groups with approximately six participants in each district. Based on each university’s response rate, four distinct groups would meet once for sixty minutes. The moderators will facilitate the group discussion and use audio and note-taking for data collection.

### 2.2. Pilot Study

To evaluate the study’s feasibility, we will conduct a pilot study for the quantitative and qualitative data to determine the administration time, clarify the instruments, and overall appearance. In addition, the pilot study will serve to identify items that may be unsuitable or misinterpreted and to gain a better understanding of the dependability and construct validity of the survey [56]. The pilot study’s goal sample size of 100 is justified by factors such as resource allocation, statistical requirements, and practicality.

The COVID Impact Scale will be translated into Greek from English. Preliminary data gathering will allow us to verify the instrument’s reliability using the Cronbach alpha test. This dependability test examines internal consistency, or how intricately connected a set of items is as a group. The preliminary results’ reliability and validity are assessed. The CIS has been translated into Spanish and Turkish, providing credible evidence [57,58]. As a result, greater evidence of CIS’s reliability and validity in numerous countries is required to improve its cultural value. To address this, the current pilot study will assess the psychometric aspects of the CIS among Republic of Cyprus university students.

### 2.3. Setting and Study Population

The target population consists of an estimated 30,000 full-time university students at 7 universities in four districts at the Republic of Cyprus, namely Nicosia, Limassol, Larnaca, Paphos. The enrolment numbers at each university vary, with the University of Nicosia having the largest enrolment with an estimated 15,000 students and the University of Central Lancashire (UCLan) Cyprus having the smallest enrolment with an estimated 1300 students. The study will employ a proportional quota sampling design. Stratification will be at the university level based on discipline (e.g., Sciences, Medicine, Humanities, etc.) and gender, with a larger number of participants from the University of Nicosia compared to smaller institutions. Expecting a 20% response rate, a sample size of approximately 2000 participants is expected to be recruited.

### 2.4. Inclusion and Exclusion

The inclusion criteria for this study will be individuals currently enrolled at the participating universities’ institutions, at least 18 years old, and either in the Bachelors, Master’s, or PhD program regardless of faculty and year of study.

The following exclusion criteria will be followed: participants under 18 years and those not currently enrolled in any university programs.

### 2.5. Data Collection

Quantitative data will be collected via an online questionnaire prepared with Google Forms and conducted in both Greek and English. The participating universities will send all registered students an explanatory email with a link to the online survey. This email will notify participants about the study’s objective, the use of their data in accordance with the General Data Protection Regulation (GDPR) and contact information for the Principal Investigator (PI) in case of any further questions. Interested participants will give digital informed permission before completing a 25 min questionnaire. At the end of the survey, participants will be asked if they want to participate in a focus group to discuss their emotional experiences post-COVID-19 in more detail.

The focus group approach will be used to collect qualitative data because its interactive aspects can stimulate a vibrant and fluid exchange of ideas and individual experiences among the students [59,60].

Structure of Focus Groups:

Each focus group will consist of approximately six students per district who are comfortable speaking English, as the discussions will be conducted in English. Four distinct groups will be formed, with each group meeting once for about 60 min. The focus groups will be moderated by members of the research team, ensuring that discussions remain on track and all participants can contribute. The focus groups will be structured around a set of guiding questions designed to elicit detailed and meaningful responses. Examples of guiding questions include the following:

- What were your primary concerns or anxieties during the COVID-19 pandemic?
- Reflecting on your interactions with professors and peers during this time, how did your relationships evolve or change?
- What notable changes did you experience in terms of resources, be it financial support or family dynamics, throughout the COVID-19 pandemic?
- Let us explore the mental health support provided by the university. Can you discuss the services or resources offered to students during the pandemic?
- How accessible did you find these mental health services, and to what extent did they meet your needs during challenging times?
- How would you personally define resilience, especially in the context of navigating the various challenges posed by the pandemic?
- From your perspective, what strategies or initiatives could universities implement to foster resilience among students, particularly during times of crisis like the COVID-19 pandemic?

All focus group discussions will be audio-recorded and transcribed verbatim to ensure data accuracy and completeness. The transcribed data will be hand coded into an Excel file. A coding framework will be created based on both the leading questions and the emerging ideas from the discussions. The data will be studied thematically. The initial coding will identify major statements and repeating ideas, which will subsequently be organized into larger themes. These themes will be improved and interpreted to reveal the data’s underlying meanings and patterns. To ensure consistency in moderation, the same set of guiding questions will be utilized in all focus groups to ensure that the themes discussed are consistent. Moderators will be trained to guarantee that discussions are conducted consistently and objectively, allowing participants to openly express their opinions. Validity: Key findings will be shared with participants for input to ensure correct interpretations based on their experiences and opinions. The focus group findings will be combined with quantitative data and current research to validate the results. Detailed descriptions of the environment and participants’ experiences will be provided to provide a thorough comprehension of the data and enhance the transferability of the findings. Using a mixed-methods approach, this study seeks to provide an extensive understanding of university students’ mental health and resilience in the post-pandemic age, utilizing both quantitative breadth and qualitative depth.

### 2.6. Research Instruments

In this cross-sectional study, we plan to use a self-designed socio-demographic questionnaire to assess each student’s demographic and social profile. This will include age, gender, relationship status, socioeconomic status (parental education, employment, and occupation), family background, religion, and previous somatic or mental health issues. In addition, academic-related questions will be asked (i.e., tuition fees, the program they are enrolled in, and year of study).

The following three scales will be utilized in the study for assessing the main variables of interest:

The COVID-19 Impact Scale (CIS) is a self-reported instrument consisting of 10 items measuring the psychological effects of the COVID-19 pandemic (e.g., “How often are you experiencing irritation regarding the COVID-19 related problems currently?” and “How much do the COVID-19 related problems interfere with your interpersonal relationships?”) [61]. All items are rated on a 5-point Likert scale, ranging from 0 (none) to 4 (very severe/very often). The internal consistency of the CIS was 0.91. This scale has been translated to Greek for this study. Permission has been granted to use the scale in the current study from the authors.

The Conner–Davidson Resilience Scale (CD-RISC) is a 25-item self-report instrument, which assesses an individual’s ability to be resilient (e.g., “I am able to deal with change”). Each rated on a 5-point Likert scale (0–4), 0 (Not true at all) to 4 (True nearly all of the time) with higher scores reflecting [62] greater resilience. Total scores range from 0 to 100. Cronbach’s alpha for the full scale was reported as 0.89, item–total correlations ranged from 0.30 to 0.70, and the test–retest reliability intraclass correlation coefficient was 0.87 [62]. This scale has been validated and translated into Greek in previous studies [62,63,64]. The owners of the scale have given their approval for the use of the scale in the present study.

The Symptom Checklist 90-Revised (SCL 90-R) is a self-report scale that measures subjective discomfort and symptomatic behavior in various dimensions of psychopathology. It consists of 90 questions which are grouped into nine major symptomatologic dimensions—subscales: Somatization (12 items), Obsessive Compulsive (10 items), Interpersonal Sensitivity (9 items), Depression (13 items), Anxiety (10 items), Hostility (6 items), Phobic Anxiety (7 items), Paranoid Ideation (6 items), and Psychoticism (10 items). Each question is graded in a 5-point Likert type (0 = none to 4 = too much). It also includes seven questions that are not included in the above subscales, related to sleep and food disorders, and they are referred to in the text as “Additional Items” and are counted on the total score. In addition to the nine subscales, three total psychopathology indicators are also exported: a) Global Severity Index (GSI), (b) Positive Symptoms Total (PST), and (c) Positive Symptom Distress Index (PSDI). The scale has been standardized in the Greek population [65] and has been used in other studies as well [65,66,67,68].Permission to employ the scale in the ongoing study has been received directly from the authors.

### 2.7. Focus Group Questions

Purpose-designed questions have been developed to guide the focus group discussion focusing on emotional experiences of participants during the pandemic (resource change, cognitive experience). At the beginning of the focus group, an icebreaker will be utilized which will be a round of introductions which would increase participant engagement early on, reducing participants remaining silent throughout the discussion. A nondirective moderating style will be used to facilitate the group to increase participation.

### 2.8. Outcomes

Primary outcomes are high or low resilience and the self-reported subjective discomfort and symptomatic behavior in various dimensions of mental health, assessed by the Symptom Checklist 90-R, namely (a) Global Severity Index (GSI), (b) Positive Symptoms Total (PST), and (c) Positive Symptom Distress Index (PSDI). The secondary outcomes are Somatization, Obsessive Compulsion, Interpersonal Sensitivity, Depression, Anxiety, Hostility, Phobic Anxiety, Paranoid Ideation, and Psychoticism.

### 2.9. Data Management

In this study, data management will entail a systematic procedure of collecting, storing, organizing, safeguarding, and evaluating both quantitative and qualitative data acquired from participants. To guarantee adequate data management, the following procedures will be taken:

Data will be collected from participants utilizing a web-based survey and focus groups will be in person. The surveys will be performed electronically through Google Forms, with replies kept on a secure server. The focus groups will be audio-recorded and verbatim transcribed to ensure anonymity in the focus groups participants will use pseudo- names in the discussion.

The obtained data will be kept on a safe, password-protected computer with limited access for the research team exclusively. The numeric data will be saved in Microsoft Excel while the qualitative data will be saved in text files, all password-protected. Data will be cleaned (checked for completeness and accuracy). Data management will be subject to the study’s ethical and regulatory standards, and the data will be de-identified before being used for analysis. Lastly, the best practices of research ethics, data, privacy, and security will be followed to ensure the integrity of the data and to protect the rights of the participants.

## 3. Data Analysis

Quantitative analysis is numerical and measurable, while qualitative data analysis is interpretation-based and relates to language, helping us to understand why certain outcomes occur [55].

Based on the research questions to be answered in this study, the following analysis will be conducted:

Descriptive statistics including mean, median, standard deviation (s.dev), and interquartile range (IQR) will be used to describe all numeric variables of interest, after their distribution is first checked (mean and s.dev will be used for normally distributed variables; otherwise, median and IQR will be used). In addition, absolute frequencies and proportions will describe the categorical variables of interest.

One-way ANOVA (analysis of variance) or independent samples *t*-test will be used to compare resilience or mental health outcomes between different demographic groups, according to the number of categories in each variable (*t*-test for binary independent variables and ANOVA for variables with >2 categories). In case of non-normally distributed dependent variables, the non-parametric equivalents (Mann–Whitney test instead of the independent samples *t*-test and Kruskal–Wallis Test instead of ANOVA) will be applied.

Correlation analysis will be used to determine crude correlations between key numeric variables of interest, (e.g., resilience and mental health outcomes). For associations between categorical variables, the chi-squared test will be used.

Multiple Linear Regression analysis will be used to assess all major associations of interest, such as that between resilience and mental health outcomes, thus answering the main study aims (1–3). In these analyses, one variable (e.g., resilience) will be treated as the main independent numeric variable of interest and the other one (e.g., depression), as the main dependent variable of interest. The model will also include specific sociodemographic factors (e.g., gender, age, area of residence, etc.,) as potential confounders, in order to adjust for these factors and present a more valid association between the independent and dependent variables of interest.

To examine aims 4–10, moderation will be incorporated into the above-mentioned regression models, by including an interaction term between the main independent variables of interest and the potential moderator(s), in turn. Sample splitting techniques such as median split, mean split, or quartile split will be utilized for moderation analysis [69]. Sample splitting can be useful in gaining insights on how socioeconomic status, social support, and demographic characteristics alter the relationships between COVID-19 impact, resilience, and mental health outcomes in the context of the aims. Furthermore, by decreasing variability within subgroups, sample splitting can improve statistical power and make it easier to identify important moderating effects. This is especially helpful when examining interactions involving several moderator factors or low sample sizes [69,70].

Structural equation modeling, specifically path analysis, will be utilized to provide a holistic picture on the complex interplay (involving moderation and mediation) between sociodemographic factors, social support, COVID-19 impact, resilience and mental health outcomes, with the latter being the ultimate outcomes of interest. This could provide a comprehensive and nuanced understanding of the relationships among these variables and help to clarify the mechanisms through which resilience, socioeconomic status, social support, and mental health outcomes are interlinked post COVID-19 pandemic.

Specific Steps for SEM Implementation.

Structural equation modeling is a linear regression framework, capable of modelling both simultaneous regression equations with latent variables. Path analysis is considered a precursor to and subset of structural equation modeling, aiming to test the hypothetical effects of a set of variables on a specified outcome variable(s), via multiple pathways, involving mediation and moderation (interaction). Path analysis is particularly useful for examining complex models leading to the outcome(s) of interest, as well as for determining which model from a set of alternative models best fits the data in statistical terms.

In the proposed study, we will use several steps to implement Structural Equation Modeling (SEM). First, we shall define the hypothesized relationships between variables in accordance with our theoretical framework. Next, during data preparation, we will handle missing data using multiple imputation (further details below) methods, as well as analyze the data for normalcy, utilizing transformations or robust estimation methods if the data are not normally distributed. We will then use the lavaan package in R to estimate the model parameters. We will evaluate model fit using indices such as RMSEA (values < 0.06), SRMR (values ≤ 0.08), chi-square test, Comparative Fit Index (CFI), and Tucker–Lewis Index (TLI). If necessary, the model will be modified based on fit indices and theoretical reasons. Validation will be carried out by splitting the sample to perform cross-validation, to ensure the model’s robustness.

Example R Code for SEM and Path Analysis

###Load necessary libraries

library(lavaan)

### Specify the SEM model

model <- ‘

###Measurement model

resilience =~ q1 + q2 + q3 + … + …

mental health =~ q4 + q5 + q6 + … + …

###Structural model

mental_health ~ resilience + socioecon_status + social_support

social_support ~ socioecon_status

resilience ~ covid_impact + socioecon_status

###Fit the model using lavaan

fit <- sem(model, data = data)

### Summarize the fit

Summary (fit, fit.measures = TRUE, standardized = TRUE)

### Assess model fit

fitMeasures(fit, c(“rmsea”, “srmr”, “cfi”, “tli”))

Multiple imputation analysis will be performed for dealing with missing data, using Multivariate Imputation by Chained Equations (MICE), performed by the relevant R package (mice). The mice package applies Conditional Multiple Imputation, which follows an iterative procedure, modeling the conditional distribution of a specific variable with missing values, based on the information of other variables. This approach will be utilized to estimate more valid regression coefficients, not influenced by missingness. In particular, *mice* creates a set number of imputed datasets that replace missing values with plausible values and the analyses are then conducted on these complete (imputed) datasets, eventually obtaining a single regression coefficient.

Qualitative analysis:

Colaizzi’s phenomenological technique will be employed in this study for qualitative analysis [59]. The following stages are included in this method: (1) reading all interview materials to form a general understanding of the research object; (2) extracting statements related to the research problem; (3) summarizing, extracting, and encoding the extracted data; (4) summarizing the encoded ideas and seeking common concepts to form themes and theme groups; (5) performing a detailed description of the relationship between the theme and the research object; (6) stating the essential structure that constitutes; and (7) returning the final analysis to the research object for verification. Two researchers will independently code, summarize, and refine the interview materials to form primary and secondary themes to ensure inter rater reliability. Discrepancies will be solved by discussions [59,71]. Finally, to ensure that our findings are accurate and credible, we will return the final analysis to the participants for verification.

To ensure reliability and validity in this study, we will employ several strategies. Inter-rater reliability will be established by having two researchers independently code, summarize, and refine the interview materials to form primary and secondary themes, resolving any discrepancies through discussion. Member checking will be conducted by sharing key findings with participants to ensure that the interpretations accurately reflect their experiences. Triangulation will be utilized by comparing qualitative findings with quantitative data and the existing literature to validate the results. Additionally, thick descriptions of the context and participants’ experiences will be provided to enhance understanding and transferability of the findings.

By utilizing both quantitative and qualitative methods, this study aims to provide a comprehensive understanding of university students’ mental health and resilience in the post-pandemic era.

## 4. Limitations and Delimitations of the Study

Selection bias is a pertinent issue to consider when conducting a study among university students, particularly in the context of mental health and resilience. The possibility of students choosing to participate in the study based on their level of engagement or pre-existing opinions about mental health could lead to non-representative samples. To address this, rigorous mitigation strategies will be implemented. High response rates will be pursued through multiple participation reminders. Also, stratified sampling techniques will ensure diverse representation across various demographics such as age, gender, socioeconomic status, and academic disciplines.

Similarly, the potential for self-report bias cannot be overlooked. Participants may overreport or underreport their mental health status and resilience due to social desirability or recall bias. To tackle this issue, the study will prioritize anonymity and confidentiality of responses, thereby encouraging honest and accurate reporting. The use of validated and reliable questionnaires will further enhance the accuracy of self-reported data.

Ensuring sample representativeness is imperative to the credibility of the study’s findings. Collaborating with multiple universities across the Republic of Cyprus and employing stratified sampling methods are key strategies to capture a broad spectrum of students. Data on demographics will be meticulously collected to assess the representativeness of the sample, and appropriate weighting adjustments will be made during the analysis phase if required.

Additionally, the study’s cross-sectional design poses another limitation. Cross-sectional studies inherently struggle to establish causality due to the inability to determine the direction of associations between variables. This limitation introduces the potential for reverse causation, wherein observed associations may not accurately reflect causal relationships. Consequently, interpretations of findings must be cautious and acknowledge the limitations inherent in the study design.

In terms of generalizability, while the primary focus of the study is on university students in the Republic of Cyprus, the potential limitations regarding the generalizability of the findings will be acknowledged. The study will explore and discuss contextual factors that might influence the results and will advocate for further research in diverse geographical and cultural settings to facilitate comparison and broader applicability of the findings.

## 5. Ethics and Dissemination

This study was approved by the Cyprus Bioethics Committee (approval no: EEC/EP/2023/31 approval date 4 December 2023). If necessary, we will obtain permission from the University of Central Lancashire (UCLan) Cyprus, Cyprus University of Technology, European University Cyprus, Neapolis University Pafos, University of Nicosia, Frederick University and University of Cyprus to conduct the study. This study will be conducted online, and all participants will be requested to reply after consenting to the research objectives.

To distribute the study’s findings, we want to submit them to a peer-reviewed journal and present them at local and international conferences to reach a broad audience. In addition, the findings of this investigation will be widely disseminated at conferences and workshops globally.

## 6. Discussion

The World Health Organization underlines the need for mental health services and recognizes the unique issues that university students encounter [72]. This study addresses these aims by investigating the resilience and mental health condition of university students in Cyprus post the COVID-19 pandemic, giving critical insights that can influence targeted treatments and support services. The findings are expected to significantly improve Cyprus’ understanding of these issues, with several key implications, including guiding targeted interventions tailored to the specific needs of university students, contributing to public health practices by providing novel insights into this demographic’s psychological well-being, and promoting resilience through programs designed to assist students in dealing with future crises.

Furthermore, the study will educate politicians and educational institutions on resource allocation and policy creation to assist student mental health. The study’s findings have the potential to significantly influence educational and mental health policies by emphasizing the need for more robust mental health services on campuses, advocating for flexible learning environments, recommending faculty and staff training programs, and encouraging curriculum changes to include mental health education and resilience-building activities. Furthermore, the study could result in greater financing for campus mental health services, emphasize the value of preventive mental health initiatives, and highlight the need for well-defined emergency response plans that incorporate mental health care during crises. Universities and policymakers should implement comprehensive mental health programs, develop crisis response protocols, promote mental health awareness and education, strengthen social support networks, adopt flexible academic policies, and encourage ongoing research into student mental health and resilience. Addressing these recommendations will enable institutions and policymakers to better help students during and after crises, resulting in a healthier, more resilient student population. The study’s findings will provide critical insights that may be used to design effective strategies and policies that prioritize and effectively support university students’ mental health and well-being.

## Data Availability

Data will be made available on request from the corresponding author.
